# A clinical study on the comparative effectiveness of micronutrient supplementation and physiotherapy in oral sub-mucous fibrosis

**DOI:** 10.6026/973206300221528

**Published:** 2026-03-31

**Authors:** Nidhi Nidhi, Sunil Sharma, Uday Narayan Sharma, M.K Sunil, Rohit Sharma

**Affiliations:** 1 Department of Oral Medicine & Radiology, NIMS Dental College & Hospital, NIMS University, Jaipur, Rajasthan, India; 2 Department of Dentistry, Nims Dental College & Hospital, NIMS University Rajasthan, Jaipur, India; 3 Department of TB & Chest, Anughar Narayan Magadh Medical College & Hospital, Bihar, Gaya, India

**Keywords:** Physiotherapy, micronutrient, oral sub-mucous fibrosis

## Abstract

Oral Sub-mucous Fibrosis (OSMF) is a chronic, progressive, potentially malignant disorder linked to areca nut chewing. Multiple therapies have been tried, but no consensus exists on the most effective approach. Therefore, it is of interest to compare the effectiveness of micronutrient supplementation and physiotherapy in managing OSMF. Patients with OSMF were randomly assigned to receive either micronutrient supplementation (Group A) or structured physiotherapy (Group B). Outcomes assessed included mouth opening and burning sensation at baseline and follow-up. Both groups showed symptomatic improvement, with physiotherapy yielding significantly greater gains in mouth opening, while both interventions reduced burning sensation. Physiotherapy is more effective than micronutrient supplementation in improving functional outcomes, though both provide symptomatic relief. Combination therapy may be beneficial.

## Background:

Oral Sub-mucous Fibrosis (OSMF) was first described by Schwartz in 1952 as "Atropica idiopathica mucosae oris" [1] and later defined by Pindborg in 1966 as an "insidious, chronic disease affecting the oral cavity and sometimes the pharynx" [2]. It is a potentially malignant disorder characterized by juxtaepithelial inflammatory response, fibroelastic changes in the lamina propria and epithelial atrophy [3]. Subepithelial and submucosal fibrosis stiffens the oral mucosa and underlying tissues, progressively restricting mouth opening and tongue movement. In advanced stages, epithelial atrophy becomes prominent and patients commonly experience burning sensation, trismus, blanching of the oral mucosa, reduced tongue mobility, taste alteration and in some cases, hearing loss due to eustachian tube involvement [4]. OSMF is a chronic, progressive condition with marked submucosal fibrosis affecting the oral cavity, pharynx and upper third of the esophagus. Clinical severity varies depending on lesion progression. The condition is strongly linked to lifestyle and cultural factors, with the highest prevalence reported in South and Southeast Asian populations. Countries such as India, Pakistan, Bangladesh, Sri Lanka, Taiwan and Southern China report high incidence. India, in particular, accounts for the largest number of global cases, with prevalence ranging from 0.2-1.2% across regions and an overall national frequency of around 0.5% [5]. As a potentially malignant disorder, OSMF carries a significant risk of malignant transformation, with estimates ranging from 3-6% [6,7]. This places it among the most serious oral precancerous conditions requiring early recognition and effective management. The etiology of OSMF is multifactorial, though a strong causal association has been established with areca nut (Areca catechu) chewing, often in the form of betel quid [8]. Case-control studies confirm that frequency and intensity of areca nut use are more critical risk factors than duration of the habit [9]. Among the nut's alkaloids, arecoline is considered the principal etiologic agent, as it alters collagen metabolism through effects on collagenases, matrix metalloproteinases andlysyl oxidases, resulting in excessive fibrosis [10].

Other contributing factors include tobacco and chilli use, nutritional deficiencies such as iron and vitamin B-complex, trace elements like excess copper and genetic predisposition. Atrophic mucosa due to poor nutrition is particularly vulnerable to chronic irritation from areca nut and chillies, accelerating disease progression [1, 12, 13 -14]. The pathogenesis of OSMF involves a complex interplay of environmental, genetic and immunological mechanisms. Betel quid, containing areca nut, slaked lime and sometimes tobacco, plays a central role [8, 9 - 10]. Areca alkaloids stimulate fibroblast proliferation and increase collagen synthesis, while copper in the quid enhances collagen cross-linking [13]. Immunological mechanisms also contribute. Yang *et al.* and Gupta and Sharma proposed that an unknown initial stimulus activates lymphocytes, leading to mast cell proliferation. Betel quid constituents further induce pro-inflammatory cytokines such as IL-6, TNF-α, IL-8 and GM-CSF, resulting in inflammatory cell infiltration and mast cell activation in the oral mucosa [15-16]. Histopathologically, OSMF is marked by excessive collagen deposition in the submucosa and progressive epithelial atrophy [3-4]. Between 10-15% of biopsied cases show epithelial dysplasia and 3-6% undergo malignant transformation into oral squamous cell carcinoma (OSCC). Data from Tata Memorial Hospital, Mumbai, indicated that nearly one-third of OSMF patients progressed to OSCC, strengthening its classification as a precancerous condition. The disease arises from multiple biological insults, with areca nut playing a central pathogenic role. The resultant imbalance between collagen synthesis and degradation, compounded by inflammatory and nutritional factors, leads to irreversible fibrosis and high malignant potential [17]. Conventional management strategies for OSMF include corticosteroids, antioxidants, micronutrient supplementation and physiotherapy [18- 19]. Among these, micronutrient therapy (iron, vitamins A, B complex, C and minerals) addresses nutritional deficiencies and improves mucosal healing, while physiotherapy reduces trismus through exercises aimed at improving mouth opening and elasticity of fibrotic tissue. Therefore, it is of interest to report the comparative improvement in clinical parameters when evaluating the effectiveness of micronutrient supplementation versus physiotherapy.

## Aim:

The present study aims to compare the effectiveness of micronutrient supplementation and physiotherapy in the management of Oral Sub- mucous Fibrosis.

## Objectives:

[1] To assess the effectiveness of micronutrients in management of oral Sub-mucous fibrosis.

[2] To assess the effectiveness of physiotherapy in management of oral Sub-mucous fibrosis.

[3] To compare the effectives of micronutrient and physiotherapy in the management of oral sub mucous fibrosis.

## Materials and Methods:

## Study design:

The present research was designed as an interventional, randomized comparative clinical study to evaluate the effectiveness of micronutrient supplementation and physiotherapy in the management of Oral Sub-mucous Fibrosis (OSMF).

## Study setting and duration:

The study was conducted in the Department of Dentistry, Anugrah Narayan Magadh Medical College and Hospital Gaya, Bihar, over a period of 12 months.

## Sample size determination:

The sample size was calculated using the formula: (see PDF)

Considering a confidence level of 95%, margin of error of 5% and population proportion of 3% with an unlimited population size, the minimum sample size was calculated as 45 per group. To account for potential dropouts, the sample size was rounded to 50 patients per group, giving a total of 100 participants.

## Selection of participants:

## Inclusion criteria:

[1] Patients with a history of tobacco/areca nut consumption in any form.

[2] Clinical features of OSMF such as decreased mouth opening, burning sensation, intolerance to spicy foods and history of vesicles/ ulcerations.

[3] Clinical diagnosis of OSMF confirmed by the examiner.

## Exclusion criteria:

[1] Patients with systemic diseases or other oral mucosal conditions.

[2] Patients already undergoing treatment for OSMF.

[3] Patients unwilling to provide informed consent or adhere to follow-up visits.

## Randomization and Grouping: (Figure 1 - see PDF)

A total of 100 eligible OSMF patients (with baseline interincisal mouth opening >20 mm) were randomly allocated into two equal groups using simple random sampling:

[1] Group A (n = 50): Received micronutrient supplementation daily.

[2] Group B (n = 50): Received physiotherapy exercises in addition to micronutrient supplementation.|

Prior to intervention, all patients received counseling regarding habit cessation.

## Intervention details:

## Group A -micronutrient supplementation:

Patients were prescribed Biostar Gold capsules once daily. Each capsule contained:

[1] Alpha lipoic acid

[2] Cyanocobalamin IP

[3] Folic acid IP

[4] Vitamin B6

[5] Chromium picolinate

[6] Selenium

[7] Zinc sulphate monohydrate

[8] Manganese sulphate USP

[9] Magnesium oxide

[10] Lycopene

[11] Flaxseed oil

## Group B - Physiotherapy + Micronutrient supplementation:

In addition to the same micronutrient supplementation, patients were instructed to perform physiotherapy exercises involving graded mouth-opening using ice-cream sticks placed between the upper and lower incisors. The number of sticks was gradually increased every 2-4 days.Compliance was reinforced with weekly recall visits to monitor adherence and provide motivation.

## Outcome assessment:

## Measurement of mouth opening:

Interincisal distance was measured between the mesio-incisal edges of the upper and lower left central incisors using a calibrated divider and ruler. Measurements were recorded in millimetres.

## Symptom evaluation:

Subjective parameters such as burning sensation, pain and intolerance to spicy foods were assessed using a Visual Analogue Scale (VAS), a 10-cm horizontal line ranging from 0 (no pain) to 10 (worst pain imaginable).

[1] 0 = No pain

[2] 1-3 = Mild discomfort

[3] 4-6 = Moderate pain

[4] 7-10 = Severe pain

## Follow-Up and evaluation timeline:

All clinical parameters (mouth opening, burning sensation, VAS score, ulceration and referred pain) were recorded at:

[1] Baseline

[2] 1 month

[3] 3 months

[4] 6 months

## Statistical analysis:

All data were entered into SPSS (Statistical Package for Social Sciences) version 21.0 (IBM, Chicago, USA) for analysis. Results were expressed as Mean ± Standard Deviation (SD) for continuous variables and as number (%) for categorical variables.

[1] Mean, Median and Standard Deviation were used for descriptive statistics.

[2] Post Hoc analysis was performed following ANOVA to compare differences between groups at multiple time intervals.

[3] A p-value <0.05 was considered statistically significant

## Ethical considerations:

The study was conducted in accordance with the Declaration of Helsinki (2013 revision). Approval was obtained from the Institutional Ethics Committee of NIMS University Rajasthan, Jaipur. Written informed consent was obtained from all participants before enrolment.

## Results:

A total of 100 patients were enrolled, with 50 participants allocated to each group. The baseline demographic characteristics such as age distribution, gender, tobacco consumption habits and smoking history were comparable between the groups. Clinical parameters, including mouth opening (measured in millimeters) and burning sensation (assessed using the Visual Analog Scale, VAS), were recorded at baseline and at subsequent follow-ups of 1 month, 3 months and 6 months. Table 1 (see PDF) presents the baseline characteristics of the study participants. A total of 100 subjects were included, with 50 in Group A (micronutrient group) and 50 in Group B (physiotherapy with micronutrients), aged between 20 and 50 years. The majority in Group A were in the 41-50 years age group (38.0%), while in Group B most participants were in the 31-40 years group (38.0%). Males predominated in both groups (78.0% in Group A and 74.0% in Group B). Tobacco consumption habits revealed that the majority used pan with tobacco (82.0% in Group A and 78.0% in Group B), followed by areca nut only (10.0% in Group A and 8.0% in Group B) and pan without tobacco (8.0% in Group A and 14.0% in Group B). With respect to smoking history, cigarette use was most common (76.0% in Group A and 56.0% in Group B), while bidi use was higher in Group B (20.0%) compared to Group A (10.0%); hookah use was relatively low in both groups (6.0% and 8.0%, respectively). A minority of participants reported no smoking history (8.0% in Group A and 16.0% in Group B). Table 2 (see PDF) shows the mean distribution of mouth opening and VAS scores at different time intervals within each study group. In Group A, the mean mouth opening improved steadily from baseline (21 ± 7.46 mm) to 28.6 ± 6.25 mm at 6 months and this change was statistically significant (F = 229.751, p< 0.01). Similarly, VAS scores increased significantly from 2.58 ± 0.67 at baseline to 5.58 ± 0.67 at 6 months (F = 600367.8, p< 0.01). In Group B, mouth opening improved from 21.96 ± 6.32 mm at baseline to 25.78 ± 5.68 mm at 6 months (F = 159.769, p< 0.01) and VAS scores increased from 2.74 ± 0.72 at baseline to 4.5 ± 0.71 at 6 months (F = 223.82, p< 0.01). These findings indicate significant improvements in both clinical parameters over time within each group.

Table 3 (see PDF) presents the post hoc analysis of mean mouth opening in Group A and Group B at different time intervals. In Group A, significant improvements were observed across all time comparisons (p< 0.01). Mouth opening increased by 3.64 mm from baseline to 1 month, 5.32 mm from baseline to 3 months and 7.6 mm from baseline to 6 months. Similarly, incremental gains were noted between 1 month and 3 months (1.68 mm), 1 month and 6 months (3.96 mm) and 3 months and 6 months (2.28 mm). In Group B, all pairwise comparisons also demonstrated statistically significant differences (p< 0.01). The increase in mouth opening was 2.1 mm from baseline to 1 month, 2.82 mm from baseline to 3 months and 3.82 mm from baseline to 6 months, with smaller but significant improvements between subsequent follow- ups. Overall, these results indicate a consistent and progressive increase in mouth opening over the 6-month follow-up period in both groups, with greater magnitude of improvement observed in Group A compared to Group B. Table 4 (see PDF) shows the inter-group comparison of mean mouth opening and VAS scores at different time intervals between Group A and Group B. At baseline, no statistically significant difference was observed in either mouth opening (21.0 mm vs. 21.96 mm, p = 0.489) or VAS scores (2.58 vs. 2.74, p = 0.225) between the groups. At 1 month, mouth opening remained comparable between the groups (p = 0.653), while VAS scores were significantly higher in Group A compared to Group B (3.56 vs. 2.92, p< 0.01). At 3 months, mouth opening again showed no significant difference (p = 0.222), but VAS scores were significantly higher in Group A (4.56 vs. 3.66, p< 0.01). By 6 months, both parameters demonstrated significant inter-group differences: Group A achieved greater mouth opening (28.6 mm vs. 25.78 mm, p = 0.02) and higher VAS scores (5.58 vs. 4.5, p< 0.01) compared to Group B.

Table 5 (see PDF) presents the intra-group comparison of mean differences in VAS scores at different time intervals within Group A and Group B.In Group A, a consistent and statistically significant improvement in VAS scores was observed across all time points. From baseline to 1 month, the mean VAS increased significantly by -0.98 units (p< 0.01). The improvement was more pronounced at 3 months (-1.98 units, p< 0.01) and reached its maximum at 6 months (-3.00 units, p< 0.01) compared to baseline. Sequential comparisons also demonstrated progressive improvement, with significant changes between 1 month and 3 months (-1.00, p< 0.01), 1 month and 6 months (-2.02, p< 0.01) and 3 months and 6 months (-1.02, p< 0.01). These findings indicate that Group A experienced a steady and continuous reduction in symptoms reflected by higher VAS scores throughout the follow-up. In Group B, although improvements in VAS scores were also statistically significant, the magnitude of change was relatively smaller compared to Group A. From baseline to 1 month, the increase in VAS was modest (-0.18, p = 0.019), but further improvements were noted at 3 months (-0.92, p< 0.01) and 6 months (-1.76, p< 0.01). Sequential comparisons also showed significant differences: between 1 and 3 months (-0.74, p< 0.01), 1 and 6 months (-1.58, p< 0.01) and 3 and 6 months (-0.84, p< 0.01). Both groups demonstrated statistically significant intra-group improvements in VAS scores over time, the magnitude of improvement was consistently higher in Group A compared to Group B, highlighting the superior effectiveness of the intervention in Group A in alleviating symptoms. Table 6 (see PDF) shows group wise comparison of distribution of subjects according to intolerance to spices at different time intervals results revealed that 96% subjects at baseline, 78% at 1 month, 48% at 3 months and 48% at 6 months intervals have intolerance to spices in group A subjects whereas 92% subjects at baseline, 83% at 1 month, 80% at 3 months and 80% at 6 months intervals have intolerance to spices in group B subjects. Statistical analysis revealed significant (P<0.01*) association at 3 months and 6 months' time intervals.

## Discussion:

Oral Sub-mucous Fibrosis (OSMF) is a chronic, progressive condition characterized by juxta-epithelial inflammation and fibrosis of the oral mucosa, often resulting in restricted mouth opening, intolerance to spicy foods, burning sensation and in advanced cases, malignant transformation. It is widely recognized as a potentially malignant disorder, with prevalence rates particularly high in India and Southeast Asia due to widespread consumption of areca nut, tobacco and related products. In the present study, the effectiveness of two conservative treatment modalities-micronutrient supplementation (Group A) and physiotherapy (Group B)-was compared with respect to improvements in mouth opening and pain reduction (VAS scores) over a period of six months. At baseline, both groups had comparable mean mouth opening (21 mm in Group A, 21.96 mm in Group B) and VAS scores (2.58 in Group A, 2.74 in Group B), with no statistically significant differences (Table 4 - see PDF). This suggests that the study population was well matched and comparable at the start, which strengthens the validity of the comparative findings. Both groups demonstrated a statistically significant increase in mean mouth opening over the study period (Table 2 - see PDF). In Group A (micronutrient therapy), mouth opening increased from 21 mm at baseline to 28.6 mm at 6 months, with consistent significant improvements at each follow-up interval (p < 0.01). Similarly, Group B (physiotherapy) showed an increase from 21.96 mm to 25.78 mm, though the magnitude of improvement was less pronounced. Post-hoc analysis (Table 3 - see PDF) confirmed that these changes were significant at all-time intervals within both groups. When inter-group comparisons were performed (Table 4 - see PDF), no difference was observed at baseline and 1 month. However, at 3 months and particularly at 6 months, Group A showed significantly greater improvement in mouth opening compared to Group B (p < 0.05). These findings suggest that micronutrient therapy yields more sustained benefits over time, while physiotherapy alone may provide short-term improvements but plateaus in effectiveness. The findings are consistent with earlier reports. Maher *et al.* and Pathak *et al.* highlighted the role of micronutrients, especially antioxidants, in improving epithelial health, reducing fibrosis and preventing disease progression [1, 2]. Similarly, Jose *et al.* demonstrated that nutritional supplementation leads to better long-term outcomes in terms of mouth opening and overall symptom relief [3]. Conversely, physiotherapy has been shown to improve mouth opening by reducing tissue stiffness and enhancing muscle flexibility (Cox *et al.* & Balappanavar *et al.*) but its success is highly dependent on patient compliance and pain tolerance. In our study, while physiotherapy was effective, the smaller gain in mouth opening compared to micronutrient therapy highlights the limitation of physiotherapy when used in isolation [4, 5].

AS scores increase significantly in groups, reflecting improvement in tolerance to pain and burning sensation (Table 2 and Table 5 - see PDF). In Group A, the mean VAS increased from 2.58 at baseline to 5.58 at 6 months, while in Group B, the increase was from 2.74 to 4.5. Intra- group comparisons showed significant improvement across all time intervals in both groups, though the degree of improvement was consistently higher in Group A. Inter-group analysis (Table 4 - see PDF) further confirmed that from 1 month onwards, Group A reported significantly better VAS scores compared to Group B, with the difference being highly significant at 3 months and 6 months (p < 0.01). This indicates that micronutrient therapy not only improved mouth opening but also provided better symptomatic relief in terms of burning sensation and tolerance to spicy foods. These results align with previous studies. Thakur *et al.* observed that physiotherapy helps in reducing pain, but patients often discontinue due to discomfort and underestimation of its importance [6]. On the other hand, micronutrients, by addressing oxidative stress and nutritional deficiencies, provide systemic benefits that translate into improved mucosal health and reduced burning sensation. The role of antioxidants such as vitamins A, C and E in neutralizing free radicals and preventing further fibrosis has been emphasized in Sinor *et al.* and Srivastava *et al.* studies [7, 8]. Previous studies done by Thakur *et al.* [20, 21] demonstrated that both micronutrient supplementation and physiotherapy significantly improve clinical parameters such as mouth opening, burning sensation, and functional limitations in patients with oral submucous fibrosis, supporting their usefulness as effective treatment modalities. These findings are in concordance with the results of the present study. Further, a meta-analysis conducted by Gopinath *et al.* [22] concluded that certain medical interventions, when combined with adjuvant non-invasive treatments, provide better clinical outcomes in the management of oral submucous fibrosis. A study by Nerkar *et al.* [23] showed that physiotherapy complemented with topical curcumin and aloe vera gel leads to greater improvement in mouth opening and reduction in burning sensation, highlighting the importance of combined non-invasive therapy. A recent study by Chitlange and Phansopkar [24] evaluated physiotherapy in oral submucous fibrosis and confirmed its effectiveness in improving mouth opening, reducing fibrosis-related functional limitations, and enhancing overall patient outcomes when structured and combined with other therapeutic interventions. The findings of this study emphasize the importance of a multimodal approach in the management of OSMF. While both physiotherapy and micronutrient therapies are effective, micronutrients appear to have a superior effect when it comes to sustained improvement in mouth opening and symptomatic relief. Physiotherapy, although beneficial, may be best used as an adjunct rather than a standalone therapy, particularly given its dependence on patient compliance and limitations in long-term outcomes. The progressive nature of OSMF and its potential for malignant transformation highlight the need for early detection and timely intervention. Conservative treatment modalities such as those studied here are particularly valuable in the early stages of OSMF, where they can halt or reverse disease progression and significantly improve quality of life. Community awareness programs targeting areca nut and tobacco cessation, along with nutritional interventions, should be integrated into preventive strategies. One limitation of this study is the relatively short follow-up period of six months. Longer-term studies are needed to assess whether the benefits of micronutrient supplementation are sustained and whether physiotherapy can achieve better results with enhanced compliance strategies. Additionally, combining both modalities may offer synergistic benefits, as suggested by Cox *et al.* and Nidhi *et al.* and this should be investigated in future trials. The results of this study indicate that while both treatment modalities improved clinical outcomes in OSMF patients, micronutrient supplementation was more effective than physiotherapy in improving mouth opening and reducing burning sensation, especially at later stages of follow-up. These findings are in line with existing literature and support the incorporation of micronutrient therapy as a primary conservative treatment for OSMF, ideally in combination with physiotherapy for optimal outcomes.

## Conclusion:

The study compared micronutrient supplementation and physiotherapy for managing Oral Sub-mucous Fibrosis (OSMF). Micronutrients significantly improved mouth opening and reduced burning sensations more than physiotherapy over six months. Patients in the micronutrient group consistently showed increased mouth opening and VAS scores, while physiotherapy had only modest, plateauing improvements reliant on compliance. The results highlight antioxidants' role in enhancing mucosal health and suggest physiotherapy is more effective as an adjunct to micronutrient therapy.
